# Simulated Annealing Based Hybrid Forecast for Improving Daily Municipal Solid Waste Generation Prediction

**DOI:** 10.1155/2014/834357

**Published:** 2014-06-30

**Authors:** Jingwei Song, Jiaying He, Menghua Zhu, Debao Tan, Yu Zhang, Song Ye, Dingtao Shen, Pengfei Zou

**Affiliations:** ^1^Key Laboratory of Digital Earth Sciences, Institute of Remote Sensing and Digital Earth, Chinese Academy of Sciences, Beijing 100094, China; ^2^Graduate School, Chinese Academy of Sciences, Beijing 100049, China; ^3^Changjiang River Scientific Research Institute, Wuhan 430010, China; ^4^Center for Geospatial Research, Department of Geography, The University of Georgia, Athens, GA 30602, USA; ^5^School of Remote Sensing and Information Engineering, Wuhan University, Wuhan 430079, China

## Abstract

A simulated annealing (SA) based variable weighted forecast model is proposed to combine and weigh local chaotic model, artificial neural network (ANN), and partial least square support vector machine (PLS-SVM) to build a more accurate forecast model. The hybrid model was built and multistep ahead prediction ability was tested based on daily MSW generation data from Seattle, Washington, the United States. The hybrid forecast model was proved to produce more accurate and reliable results and to degrade less in longer predictions than three individual models. The average one-week step ahead prediction has been raised from 11.21% (chaotic model), 12.93% (ANN), and 12.94% (PLS-SVM) to 9.38%. Five-week average has been raised from 13.02% (chaotic model), 15.69% (ANN), and 15.92% (PLS-SVM) to 11.27%.

## 1. Introduction

Municipal solid waste (MSW) often refers to the discarded materials like trash or garbage produced from daily life in urban areas. However, as a result of the high-speed urbanization process and dynamic population fluctuation in recent years, large amounts of MSW have been generated and have led to environmental pollution and health problems [1–3]. Therefore, an efficient and accurate MSW management system is essential for the planning and management of the entire urban systems, even though difficulties exist due to the high complexity and uncertainty of MSW amount. Specifically, an accurate prediction of MSW generation is a crucial and fundamental process in solid waste management system as it provides guidance for transport and disposal resources allocation. Among the large amount of proposed MSW prediction methods, time series analysis is an effective method for determining the temporal trend from history data and their own patterns [4]. Compared with other multivariable models involving demographic, socioeconomic, and other explanatory variables, the time series analysis method is more flexible and feasible due to its convenient manner of data acquisition.

To date, a number of research efforts have explored effective methods in MSW generation prediction utilizing time series analysis. Conventional statistical models have been widely used since the 1970s. Linear time series models, such as the seasonal autoregressive and moving average (sARIMA) model, have been used by Navarro-Esbri et al. [5] and Xu et al. [6] to simulate and predict the MSW time series data. Other studies treated MSW generation as a nonlinear complex system and introduced machine learning methods to mine the past patterns and to make prediction. Grey system theory [1, 6, 7], artificial neural network [8, 9], and support vector machine [10–14] are most widely used for monthly and weekly MSW generation predictions. These algorithms used a stochastic model to determine the optimum black-box model in training process and apply it to further prediction. There are other sorts of algorithms, however, using nonlinear dynamic method in long term MSW prediction. Navarro-Esbri et al. [5] proposed a nonlinear dynamics algorithm by state space reconstruction and a fitting function for this trajectory of the system. Song and He [15] proposed a chaotic model of nearest neighbor state space reconstruction for multistep ahead MSW generation prediction. These nonlinear dynamic system methods are based on the embedding theorem of Takens [16] and aim to rebuild the dynamic behavior from a discrete time series data with time delay and embedding dimension.

Hybrid forecast or hybrid model has long been used in power load [17, 18], economics [19, 20], hydrology [21, 22], and many other fields. However, the potential of hybrid models for MSW generation prediction has seldom been developed. Xu et al. [6] have proposed a fixed weighted hybrid forecast method integrating sARIMA and grey system theory (GM (1, 1)). However, a fixed weighted hybrid forecast is only suitable for medium and long term prediction because the model performance is changing constantly. At the time of this writing, there was still no report of using variable weighted hybrid forecast in daily MSW generation forecasts. In this study, a simulated annealing (SA) based variable weighted hybrid method was proposed to combine the chaotic model, ANN model, and partial least square support vector machine (PLS-SVM) model for daily municipal solid waste prediction and the results showed that the hybrid forecast outperformed the three individual models. Our study results also showed that the hybrid forecast is more accurate in comparison with traditional forecast methods like chaotic model, ANN, or PLS-SVM.

In this paper, Section 2 introduces the basic theory of the SA based hybrid model and three candidate models: ANN, PLS-SVM, and the chaotic model.  Section 3 compares the time series modeling results between the hybrid model and three individual models, discusses the accuracy and stability of the proposed method, and summarizes the pros and cons. Finally, Section 4 provides the main conclusions of the study.

## 2. Material and Methods

### 2.1. Data

Daily MSW garbage generation data were obtained from the Seattle Public Utilities website. The data consisted of up to 1001 consecutive days ranging from January 2011 to September 2013. The whole dataset was split into three parts: first 851, middle 50, and last 100. The first 851 values were used for the three model training, the middle part was used for deciding initial weights, and the last 100 values were used as comparison to evaluate the model performances.

### 2.2. SA Based Hybrid Model

As the real world is often complex in nature, no individual model can fit all conditions when conducting predictions. Instead, hybrid forecast methods can compensate the errors caused by using one single model and make more precise predictions in an extent [20]. Considering the different performances and accuracies of individual models, variable weighted hybrid forecast method based on history performances will make full use of all integrated models and conduct better predictions [23]. In this study, SA method was adopted to determine the weights for each forecast step.

The SA based hybrid forecast weighs individual forecast methods based on their prior model performances. As each model is stable in a short term, the best weight combination for the past time window will be optimum for some time. As formula (1) shows, the hybrid model forecast (*F*) is composed of *k* individual model forecasts with corresponding weights. Optimum linear weight assignment is carried out through SA which we will describe later. Consider

(1) $$\begin{array}{c} F=\overset{k}{\underset{i=1}{\sum }}\omega_{i}m_{i}. \end{array}$$

**Figure 1:**
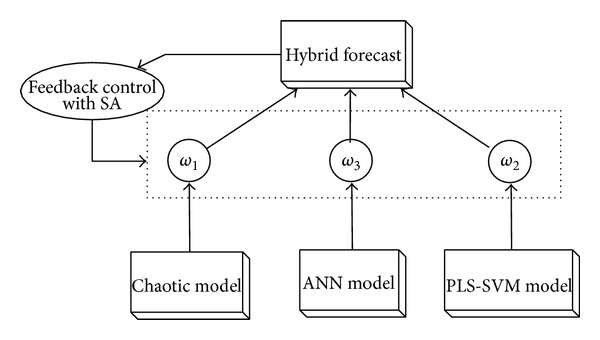
Structure of the hybrid model. The weights are decided by feedback in the SA process to optimize the best forecast of a time window.

Figure 1 provides the basic structure for the SA based hybrid model. Note that there is a time window for picking the training data for the optimizer. This is because the MSW system is highly susceptible to its environment such as economics or weather. Prior to date observations may contain different patterns which can influence each individual model differently. As urban areas do not change dramatically, an appropriate time window will provide relevant training data for the optimizer to determine the best weight combination.

Developed in 1983, simulated annealing is a heuristic algorithm for optimization [24]. It has been widely used in a broad range of application areas ever since then. Based on thermodynamics, this method is controlled by a sequence of Markov chains by gradually decreasing the temperature of the system. In this paper, all the weights form a state vector and SA searches the optimum one in the state space by applying random generated state to training data. For any pair of the states *i* and *j*, the probability of the system moving from state *i* to state *j* is denoted by *G*_*i*,*j*_(*t*) and the probability of acceptance is denoted by *A*_*i*,*j*_(*t*). *G*_*i*,*j*_(*t*) of each neighbor state *j*, as in the domain of *N*(*i*), will be selected with inverse distance possibility. The possibility of inferior state acceptance is exp⁡⁡((*f*(*i*) − *f*(*j*))/*t*). This equation indicates that (1) the worse the state *j* is, the less likely that it will be accepted. (2) As temperature *t* declines, the possibility of inferior state acceptance becomes less and less. As Dowsland and Thompson [25] point out, the performance of this algorithm is largely dependent on the cooling schedule which is the annealing rate of temperature (*t*). Consider

(2) $$\begin{array}{c} P_{i,j}\left(t\right)=\{\begin{array}{cc} G_{i,j}\left(t\right)A_{i,j}\left(t\right) & \text{if}\;\;i\neq j,\\ 1-\underset{l\in S,\;l\neq i}{\sum }G_{i,l}\left(t\right)A_{i,l}\left(t\right) & \text{if}\;\;i=j, \end{array} \end{array}$$

where

(3) $$\begin{array}{c} G_{i,j}\left(t\right)=\{\begin{array}{cc} \frac{1}{\left|N\left(i\right)\right|} & \text{if}\;\;j\in N\left(i\right),\\ 0 & \text{otherwise}, \end{array}\;\text{for}\;\text{any}\;t,\\ A_{i,j}\left(t\right)=\{\begin{array}{cc} 1 & \text{if}\;\;f\left(j\right)\leq f\left(i\right)\\ \exp\left(\frac{\left(f\left(i\right)-f\left(j\right)\right)}{t}\right) & \text{otherwise}. \end{array} \end{array}$$

### 2.3. A Univariate Chaotic Model

First proposed by Packard et al. [26] and Takens [16], phase space reconstruction is a technique to rebuild the nonlinear dynamic system by using a time delay and embedding dimension for a discrete time series. For a time series *x* = {*x*_1_, *x*_2_, *x*_3_,…, *x*_*n*_} with length *n*, the phase space coordinate can be expressed in the form of vectors *X* = {*X*_1_, *X*_2_, *X*_3_,…, *X*_*N*_}, where length *N* = *n* − (*m* − 1)*τ*; *m* denotes the embedding dimension; *τ* denotes the time delay. The coordinate of each point in phase space is

(4) $$\begin{array}{c} X_{i}=\left\{x_{i},x_{i+\tau },x_{i+2\tau },\ldots ,x_{i+(m-1)\tau }\right\}. \end{array}$$

In this way, the univariate time series is constructed as a multivariate time series and its future status can be predicted by analyzing the evolution of this dynamic system. One of the most commonly used analysis method is the local forecast approach. Based on the self-similarity of chaotic attractor in which the current point is similar to the trajectories of its neighboring points, it searches several neighboring points and makes forecast by averaging the next points of these searched points. In this method, calculate the distance of the *k*th selected point to the current point *d* ^*k*^. Assign each point to the weight value by *w*_*n*_^*k*^ = *e* ^−(*d*^*k*^/*d*_min⁡_)^, where *d*_min⁡_ is the minimum distance. *m* step forecast is the weighted average of the *n* step ahead neighboring point $\overset{-}{X_{n+m}}=(w_{n}^{k}*X_{n+m}^{k})/\sum_{k=1}^{p}w_{n}^{k}$, where *X*_*n*_^*k*^ is the *k*th point to the current point and *X*_*n*+*m*_^*k*^ is the *m* step ahead of the *k*th point.

### 2.4. Artificial Neural Network

**Figure 2:**
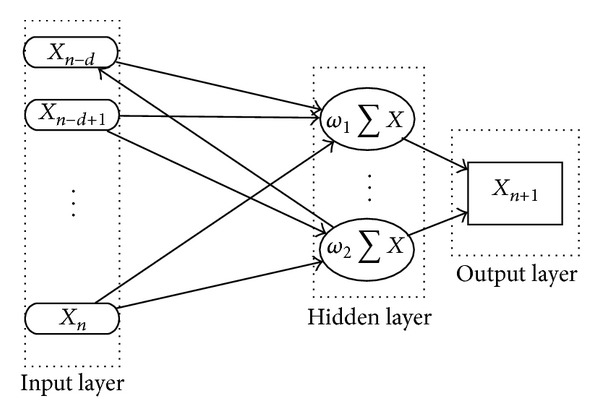
Basic structure of ANN forecast model.

Zade and Noori [8] first adopted a multilayer perceptron with back propagation for predicting MSW generation.  Figure 2 shows the three-layer neural network (input layer, hidden layer, and output layer) with feed forward structure including back propagation of error.

The input of this network is a time window of *d* observations from *X*_*n*−*d*_ to *X*_*n*_. The output of the network is predicted result *X*_*n*+1_. In hidden layer,

(5) $$\begin{array}{c} {\text{Net}}_{j}=f_{\text{sig}}\left(\overset{n}{\underset{i=n-d}{\sum }}v_{ij}X_{i}\right), \end{array}$$

where Net_*j*_ represents the *j*th node in the hidden layer and *v*_*ij*_ represents the activation function of a node. This study adopts sigmoid function used by Zade and Noori [8] as follows:

(6) $$\begin{array}{c} f_{\text{sig}}\left(x\right)=\frac{1}{1+\exp\left(-ax\right)}, \end{array}$$

where *a* is a constant. In the output layer, the prediction is calculated through the following function:

(7) $$\begin{array}{c} \text{HSI}=f_{0}\left(\overset{n}{\underset{j=0}{\sum }}\omega_{j}{\text{Net}}_{j}\right), \end{array}$$

where *ω*_*j*_ is the corresponding weight for each hidden node and *f*_0_ is the activation function. In this study, we use the commonly used line function. Both *ω*_*j*_ and *v*_*ij*_ are assigned with random values initially and are then adjusted by the delta rule derived from the learning samples.

### 2.5. Partial Least Square Support Vector Machine

Originally proposed by Wold [27], partial least square (PLS) assumes that *X* is an *n* × *p* matrix and *Y* is an *n* × *q* matrix. To build a PLS model, *X* is regressed onto the *x*-scores (*T*) to predict the *y*-scores (*U*); then *T* and *U* in turn are used to predict the responses *Y*. As a result, *X* and *Y* can be expressed as *X* = *TPT* + *E* and *Y* = *UQT* + *F*, where *Pp* × *r* is *X* loadings, *Q*1 × *r* is *Y*-loadings, *Tn* × *r* is *X*-scores, *Un* × *r* is *Y*-scores, *En* × *p* is *X*-residuals, and *Fn* × 1 is *Y*-residuals.

The details of the underlying principle of support vector machine (SVM) have been well described in literature (Vapnik et al. [28]; Vapnik [29]). It assumes that the relationship between the dependent and independent variables is given by a deterministic function *f*(*x*):

(8) $$\begin{array}{c} f\left(x\right)=\left(\omega \cdot \Phi \left(x\right)\right)+b, \end{array}$$

where *ω*, Φ, and *b* represent a nonlinear transformation from *R*_*n*_ to high dimensional space.

The goal of SVM is to find the value of *ω* and *b* so that values of *x* can be determined by minimizing the regression risk:

(9) $$\begin{array}{c} R_{\text{reg}}\left(f\right)=C\overset{l}{\underset{i=0}{\sum }}\Gamma \left(f\left(xi\right)-yi\right)+\frac{1}{2}{||\omega ||}^{2}, \end{array}$$

where *C* is a constant, Γ(·) is a cost function, and vector *ω* can be written in terms of data points as

(10) $$\begin{array}{c} w=\overset{l}{\underset{i=1}{\sum }}\left(\alpha i-{\alpha i}^{*}\right)\Phi \left(xi\right). \end{array}$$

Substitute (10) into (8), the generic equation can be rewritten as

(11) f(x)=∑i=1l(αi−αi∗)(Φ(xi)·Φ(x))+b=∑i=1l(αi−αi∗)k(xi,x)+b.

The dot product can be replaced with the kernel function *k*(*xi*, *x*). The *ε*-insensitive loss function is the most widely used cost function [30]. The function is in the following form:

(12) $$\begin{array}{c} \Gamma \left(f\left(x\right)-y\right)=\{\begin{array}{cc} \left|f\left(x\right)-y\right|, & \text{for}\;\;\left|f\left(x\right)-y\right|\geq \varepsilon \\ 0 & \text{otherwise}. \end{array} \end{array}$$

The regression risk and the *ε*-insensitive loss function can be minimized by solving the quadratic optimization problem in the following:

(13) 12∑i,j=1l(αi−αi∗)(αj∗−αj)k(xi,xj)  −∑i=1lαi∗(yi−ε)−αi(yi+ε).

Subject to

(14) $$\begin{array}{c} \overset{l}{\underset{i=1}{\sum }}\alpha i-{\alpha i}^{*}=0,\;\alpha i,{\alpha i}^{*}\in \left[0,C\right], \end{array}$$

where *αi* and αi* are Lagrange multipliers. The variable *b* can be computed by applying Karush-Kuhn-Tucker (KKT) conditions.

## 3. Results and Discussion

To assess the performance of the hybrid model and the three individual models, three indices for error prediction were calculated: mean absolute percentage error (MAPE), root mean square error (RMSE), and correlation coefficient (*R* ^2^).

MAPE is a commonly used index that measures the accuracy as a percentage in time series forecasting. It is defined as follows:

(15) $$\begin{array}{c} \text{MAPE}=\frac{1}{n}\overset{n}{\underset{t=1}{\sum }}\frac{R_{t}-F_{t}}{R_{t}}, \end{array}$$

where *R*_*t*_ is the observation and *F*_*t*_ is the forecast.

RMSE is frequently used to indicate the sample standard deviation of the forecast and observation. It is defined as follows:

(16) $$\begin{array}{c} \text{RMSE}=\sqrt{\frac{\sum_{t=1}^{n}{\left(R_{t}-F_{t}\right)}^{2}}{n}}. \end{array}$$

Coefficient of determination (*R* ^2^) is a measure of linear correlation of two variables. It indicates how well the observation and forecast values fit a line. Consider

(17) $$\begin{array}{c} R^{2}=1-\frac{\sum_{t=1}^{n}{\left(F_{t}-R_{t}\right)}^{2}}{\sum_{t=1}^{n}{\left(R_{t}-\overset{-}{R}\right)}^{2}}, \end{array}$$

where $\overset{-}{R}$ is the mean values of *R*_*t*_.

In this study, we adopted three commonly used models as individual models: chaotic model, ANN, and PLS-SVM. The embedding dimension, time delay, and nearest neighbor values of the chaotic model are estimated using heuristic analysis to be 7, 4, and 9. Since previous literature reported that an advanced ANN model nonlinear autoregressive with exogenous input (NARX) outperforms standard neural network based predictors [31, 32], NARX is employed as a substitution to ANN model. Time delays of both ANN and PLS-SVM models were also evaluated as 7 according to heuristic analysis. This is consistent with 7 days a week as daily MSW is a pseudoperiodic series. After training the three models with first part of data (1 to 851-*n* + 1), *n*-step ahead predictions were carried out in the second part (852-*n* + 1 to 900-*n* + 1). In order to obtain reliable weights, SA based weight estimation was implemented from 901-*n* + 1 data prediction. In each prediction step, ANN and PLS-SVM models were implemented for 5 times and a medium performance was recorded for further hybrid forecast to avoid nonstable results caused by random walk involved in these models. In SA hybrid forecast, decay scale, step factor, and tolerance are set to 0.95, 0.02, and 1.0 × 10^−9^.

**Figure 3:**
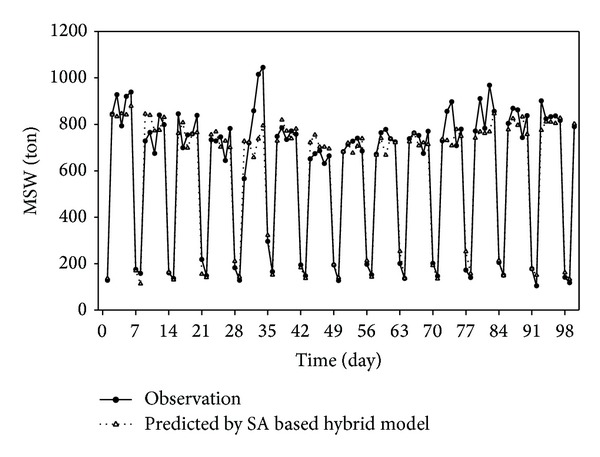
Forecast results by SA based hybrid model of one step ahead prediction. Time span covers the last 100 days.

**Figure 4:**
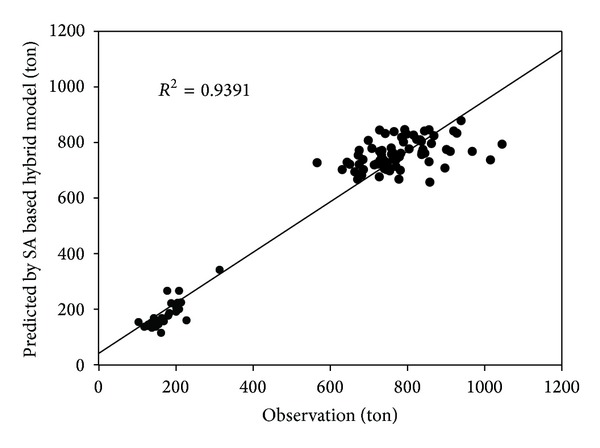
*R* ^2^ of observation and SA based hybrid forecast of one step ahead prediction. Time span covers the last 100 days.

Figures 3 and 4 illustrate the performance of SA based hybrid model in one step ahead prediction and its corresponding correlation coefficient. In one step ahead prediction, the average MAPE is lowered from 10.08% (chaotic model), 11.47% (ANN model), and 13.30% (PLS-SVM) to 8.80%. Only 8 days exceed 20% and one day exceeds 40%. These results show that the SA based hybrid model has better forecast ability than any individual model and can be further applied to daily prediction. Note that the high *R* ^2^ in Figure 4 results from the huge gap between workday (Monday to Friday) and weekend (Saturday and Sunday). If we calculate *R* ^2^ for these two different patterns separately, we will get a much lower result (0.518 for weekday and 0.656 for weekend). The MAPE and *R* ^2^ of the results are inferior to weekly MSW forecast prediction in previous studies [12, 13] due to the fact that daily MSW is more likely to be influenced by random chance.

**Figure 5:**
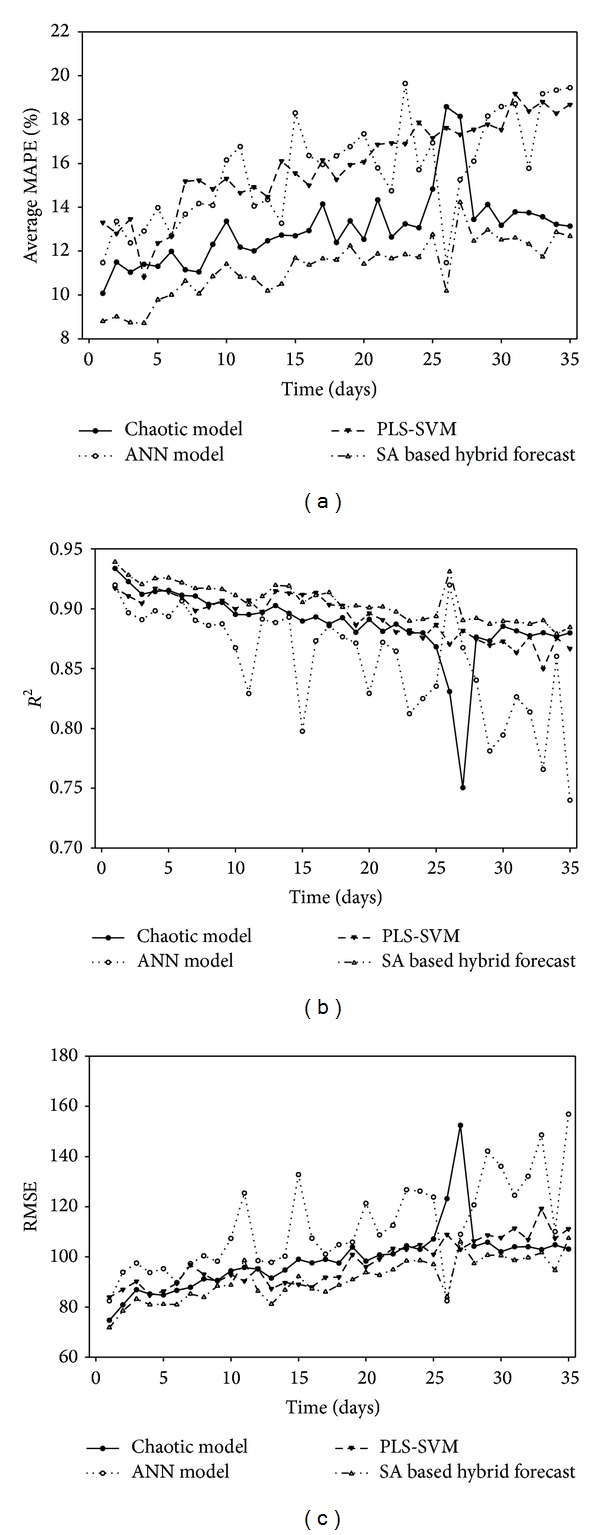
Multidays ahead prediction ability (35 days). (a) Average MAPE. (b) *R* ^2^. (c) RMSE.

To test the multistep ahead forecast ability of the hybrid model, we implemented all the models from 1 day to 5 weeks ahead (Figure 5). All three indices indicate that the SA based hybrid forecast outperforms the three individual models, meaning that it can be recommended for practical use. Previous report [15] has shown that chaotic model outperforms the other two models around 2%. In this study, the hybrid model raises the accuracy of chaotic model to another 1.75%.

Different from weekly MSW generation [8, 9, 12, 13], daily data fluctuates quasiperiodically regarding 7 days a week which is also equivalent to time delay estimated from the three individual models. In this paper, we calculated one week average MAPE and RMSE from the first week to the fifth week and measured the degradation of each model shown in Tables 1 and 2. The degradation is estimated by the average difference of each neighboring week as in the following formula:

(18) $$\begin{array}{c} \text{Degradation}=\frac{1}{4}\overset{4}{\underset{i=1}{\sum }}\left|{\text{Param}}_{i+1}-{\text{Param}}_{i}\right|, \end{array}$$

where Param_*i*_ can be either MAPE or RMSE or MAPE of the *i*th week.

Both tables suggest that SA based hybrid model not only has better forecasts, but also degrades less than the three individual models. This means that the hybrid model is more suitable and rigorous in multistep ahead prediction. We accumulated the three weights for each model in one step ahead prediction and found that the weights assigned to each model are 0.43 (chaotic model), 0.24 (ANN model), and 0.32 (PLS-SVM model). This is consistent with the accuracy shown in Tables 1 and 2. Comparing performances of three individual models in Figure 5, the chaotic model performs better than the other two models and ANN model performs the worst. The different weights for the three models are also in accordance with the three different performances. If a very bad performance model is involved in hybrid forecast, it will be assigned very low weight which makes it useless in hybrid forecast.

## 4. Conclusions

With the rapid process of urbanization, a growing amount of municipal solid waste is produced in daily life. Aiming at solving accurate prediction of MSW generation for policy making and optimum resources allocation, this paper proposed a simulated annealing based variable weighted hybrid forecast method for combining the chaotic model, ANN model, and PLS-SVM model to improve multistep MSW generation forecasts. By applying this SA based hybrid method to candidate models, more accurate forecasts can be produced. The results of the three indices (MAPE, RMSE, and *R* ^2^) show that the SA based hybrid forecast not only has better prediction capabilities, but also degrades less when applied to longer forecasts.

Previous studies have utilized hybrid models to improve the prediction performances of weekly or monthly time series MSW data for midterm and long term forecasting [6, 10, 11]. However, these studies simply choose the weights beforehand using various disciplines, which cannot choose the best weight combination automatically during the prediction process. Our study integrates the SA method to determine the weights dynamically, which can make full use of all 3 models, and proves to have improved the prediction performances of existing models well. Yet this study is just a preliminary exploration for integrating variable weighted models into daily MSW prediction. Practically, a hybrid model should be tested on historical data first before applying it to real data prediction. More studies will be conducted to explore different variable weighted models to support the time series prediction in solid waste area.

## Figures and Tables

**Table 1:** Comparison of three models and SA based hybrid forecast. Forecast efficiency degrading means the average degradation of the neighboring week in MPAE.

	Chaotic model	NARX	PLS-SVM	SA based hybrid model
Predictability (MAPE, 1st week average)	11.21%	12.93%	12.94%	9.38%
Predictability (MAPE, 2nd week average)	12.30%	14.69%	15.08%	10.65%
Predictability (MAPE, 3rd week average)	13.20%	16.69%	15.84%	11.69%
Predictability (MAPE, 4th week average)	14.85%	15.70%	17.34%	12.12%
Predictability (MAPE, 5th week average)	13.54%	18.46%	18.38%	12.52%
Forecast efficiency degrading (MAPE)	1.24%	1.88%	1.36%	0.79%

**Table 2:** Comparison of three models and SA based hybrid forecast. Forecast efficiency degrading means the average degradation of the neighboring week in RMSE.

	Chaotic model	NARX	PLS-SVM	SA based hybrid model
Predictability (RMSE, 1st week average)	83.81	92.79	88.32	80.21
Predictability (RMSE, 2nd week average)	93.31	103.96	91.21	87.65
Predictability (RMSE, 3rd week average)	99.41	111.69	93.79	90.16
Predictability (RMSE, 4th week average)	113.61	114.46	104.30	96.55
Predictability (RMSE, 5th week average)	103.16	132.74	108.72	99.23
Forecast efficiency degrading (RMSE)	10.06	9.99	5.10	4.75
